# Optimal Design of Bubble Deck Concrete Slabs: Sensitivity Analysis and Numerical Homogenization

**DOI:** 10.3390/ma16062320

**Published:** 2023-03-14

**Authors:** Natalia Staszak, Tomasz Garbowski, Barbara Ksit

**Affiliations:** 1 Doctoral School, Department of Biosystems Engineering, Poznan University of Life Sciences, Wojska Polskiego 28, 60-637 Poznań, Poland; 2 Department of Biosystems Engineering, Poznan University of Life Sciences, Wojska Polskiego 50, 60-627 Poznań, Poland; 3 Institute of Building Engineering, Poznan University of Technology, Piotrowo 5, 60-965 Poznań, Poland

**Keywords:** hollow concrete slab, numerical homogenization, optimal design, bubble deck slab, sensitivity analysis

## Abstract

The use of layered or hollow floors in the construction of buildings obviously reduces the self-weight of the slab, and their design requires some expertise. In the present work, a sensitivity analysis and numerical homogenization were used to select the most important characteristics of bubble deck floors that have a direct or indirect impact on their load capacity. From the extensive case study, conclusions were drawn regarding the optimal selection of geometry, materials, and the arrangement and size of air voids in such a way as to ensure high stiffness of the cross-section and at the same time maximally reduce the self-weight of the slabs. The conducted analyses showed that the height of the slab and the geometry of the voids had the greatest impact on the load-bearing capacity. The concrete class and reinforcement used are of secondary importance in the context of changes in load-bearing capacity. Both the type of steel and the amount of reinforcement has a rather small or negligible influence on the bubble deck stab stiffness. Of course, the geometry of the voids and their arrangement and shape have the greatest influence on the drop in the self-weight of the floor slabs. Based on the presented results of the sensitivity analysis combined with numerical homogenization, a set of the most important design parameters was ordered and selected for use in the optimization procedure.

## 1. Introduction

The history of ceilings as a covering dates back to the Stone Age. It was during the Paleolithic era that the vaulting of rocks was used as a barrier against atmospheric agents such as precipitation or solar radiation. As early as 5450–4400 BC, Egyptian civilization used palm trunks stacked tightly next to each other as a supporting structure for the vault. Additionally, in ancient Greece, stone architraves were used as support structures for wooden beams and were covered with planks or terracotta slabs [1,2]. The further evolution of stone ceilings in Greece contributed to the development of coffered ceilings. During subsequent eras, other materials began to be used to make different coverings.

The beginning of documented knowledge of reinforced concrete is generally considered to have occurred in 1867, when the Frenchman Joseph Monier patented a concrete pot reinforced with wire mesh. A few years later, in 1892, Johann Franz Kleine, a master mason from Essen, patented a ceiling consisting of steel beams and an infill of ceramic slabs reinforced with cooper [3]. With the beginning of the 20th century, structural thought developed and ceilings with concrete slabs on steel beams and ceilings with monolithic reinforced concrete beams appeared. The constant search for new structural solutions to meet innovative architectural visions led to the development of monolithic prestressed floors [4,5,6]. Due to the greater strength of the floors made of these materials, the design and weight was modified to meet the needs of the new large-scale spaces. In order to reduce the dead weight of the structure, in the late 1990s, Jorgen Bruenig designed a deck slab with plastic hollow bubbles in the center of the structural element. The new design was intended to eliminate concrete, which has no structural function, and thus reduce the dead weight of the slab [7,8,9,10,11,12].

The idea of ceiling technology has remained unchanged in its general concept for years. Every floor should meet certain requirements whether revitalizing a building or building a new one, and the design requirements determine the choice of technology and construction [13,14]. These requirements can be divided into technical and economic requirements. Technical requirements relate to strength, stability, stiffness, thermal insulation, sound insulation, durability, and fire resistance. The economic requirements can include, in particular, the low construction and operating costs of the proposed development. For structures covering large spaces, increasing the span and reducing the weight is an important consideration [15]. The bubble deck ceiling is a modern version of the box floor created during the Renaissance. In the bubble deck ceiling, superfluous concrete mass is removed from the structure while the two-way action of the element is not altered. The result is a robust structure made of interlocking quasibeams, between which a plastic infill is placed in the space created. This filling is realized by hollow spheres or lozenge-shaped lumps made of HDPE (High-Density Polyethylene), depending on the designed floor thickness. To prevent the displacement of the spheres during concreting, they are secured with a special steel bar basket and placed between the lower and upper reinforcement of the reinforced concrete slab [16,17,18]. Another variation in the floor with the concrete removed in the web is the Cobiax floor. This floor differs from the bubble deck in that the latter has a large-dimensional spatial structure and the modified floor consists of independent baskets with spheres (see Table 1).

According to researchers [7,8,9,10,11,12,17,18,19,20,21,22,23], ceilings with HDPE inserts have significantly reduced mass. Concrete usage is reduced as 1 kg of recycled plastic replaces 100 kg of concrete. Thus, the dead weight is reduced by up to 50% [7,8]. The bubble diameter varies between 180 mm to 450 mm. Depending on this, the slab depth is 230 mm to 600 mm, where the distance between the bubbles must be greater than 1/9th of the bubble diameter. The bubble used can be spherical or ellipsoidal in shape [8,12,18]. As studies have shown [23], the bubble deck slabs reduce the amount of concrete by 33% and reduce the price by 30% compared to conventional solid slabs [22].

This makes it possible to achieve single-span spans of up to 18 m–20 m, with a required floor thickness of approx. 60 cm in multispan systems. In addition to its many advantages, this modern floor construction solution fits in perfectly with the idea of sustainable, ecological construction, as the HDPE plastic used for the inserts is considered by many researchers [10,11,24] to be the best type of plastic as it is the safest for health and can be recycled and used, for example, to produce bubbles of other shapes.

**Table 1:** Differences between floors with HDPE inserts [25].

Bubble Deck	Cobiax
One spatial structure, upper and lower reinforcement combined	Single baskets with balls combined with only one (upper or lower) steel mesh
Reinforcement to match the cartridges (balls)	Bottom and top reinforcement independent of inserts (balls)
Plate thicknesses 25–60 cm	Thicknesses from 20–60 cm
There is a danger of damaging the bullets	No danger of damaging the bullets

Such ceilings are usually designed in an analytical manner using the available standards and guidelines contained in manufacturers’ catalogs. However, this approach is very time consuming and impractical because, in most cases, the engineering solution must be obtained quickly and must be repeatable for different load schemes and structures. Additionally, the use of full three-dimensional FE models of such floors in analyses involves a lot of work and is uneconomical. The solution to the above problem may be the use of simplified models obtained as a result of homogenization.

The homogenization technique allows scholars to replace the complex multilayer composite cross-section with a model with equivalent parameters. Therefore, the behavior of the replacement model is very similar to the initial (reference) model. Issues related to the homogenization of complex cross-sections of structures have been raised by many researchers for several decades. It is possible to distinguish, e.g., the method of periodic homogenization presented by Baunnic [26], the method based on an inverse analysis [27], or the homogenization method based on strain energy [28]. The differences between static and dynamic homogenization are presented in [29]. On the other hand, a list and comparison of different homogenization methods for sandwich and composite panels with a corrugated core can be found in [30]. Xin et al. proposed an approach based on the mechanics of the genome structure [31,32]. Biancolini proposed the homogenization method based on equivalent strain energy [33], in 2021, this method was improved by Garbowski and Gajewski [34]. Homogenization based on strain energy equivalence is a universal method that can be used not only for cardboard homogenization. It was used, e.g., for the homogenization of the cross-section of prefabricated Filigran slabs [35] and the analysis of thin-walled beams [36].

This paper presents the particular application of the numerical homogenization method based on the equivalence of strain energy between the full 3D RVE model and the simplified model of a single-layer coating. Initially, the method was used only for the analysis of shell structures [33,34]. This paper presents the application of the method to solid elements with internal and regular holes as well as truss elements. The application of the homogenization technique is shown on the example of a bubble deck ceiling with evenly spaced openings over the entire surface. The adopted homogenization method was used here together with a standard sensitivity analysis to automate the optimization of the numerical model of bubble deck reinforced concrete slabs. The presented methodology allows scholars to easily define both the objective function and fully automatically control all the design parameters of such ceilings. The novelty of the presented work is the algorithm, which was prepared to automatically optimize any reinforced concrete ceiling with regular openings, and the original homogenization method (adapted to the problem analyzed in this paper), which significantly improves the process of optimally designing ceilings constructed with bubble deck slabs.

## 2. Materials and Methods

### 2.1. Representative Volume Element

The numerical examples considered in this article were reinforced, concrete bubble deck slabs. The analyses were carried out for two selected versions of the above ceiling (BD340 and BD230) using the numerical homogenization method, the details of which are described in Section 2.2. The influence of particular floor slab parameters on the change in its stiffness and weight was examined. For this purpose, it was necessary to correctly define the stiffness matrix RVE (representative volume element) of the floor element. The RVE was created as a result of separating a repetitive fragment of the structure from the entire 3D plate model.

**Figure 1:**
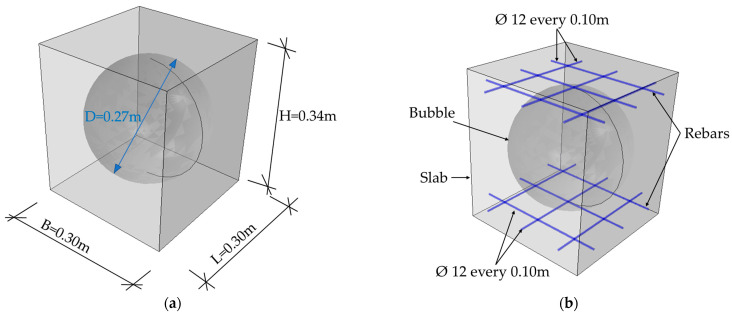
RVE-1—BD340: (**a**) geometry; (**b**) rebars.

In the first model, a typical BD340 bubble deck floor was analyzed. The basic RVE of the slab was 0.34 m high, 0.30 m wide, and 0.30 m long, see Figure 1a. The width and length were adopted in such a way as to maintain the minimum required distance between the bubbles for this type of ceiling, which was 0.03 m. The void was of a spherical shape with a diameter of 0.27 m. The slab was modeled as a solid 3D structure made of concrete. The bidirectional reinforced plate had a degree of bending reinforcement of 0.66% in both directions. This gave the reinforcement mesh the ability to move in two directions, up and down, and the mesh consisted of bars with a diameter of ϕ=12 mm spaced every 0.10 m, see Figure 1. These elements were modeled as steel wire structures embedded in the concrete slab.

In the model, the concrete cross-section was divided into C3D10 solid elements, i.e., 10-node quadric tetrahedron elements with three degrees of freedom with dimensions of 0.05 m in each RVE direction. The steel reinforcement was meshed with truss elements T3D2 (2-node truss elements with three degrees of freedom according to [37]). The discretization of the BD340 model gives a total number of 5289 elements and 9198 nodes, which translates to 27,594 degrees of freedom (since all the elements have three degrees of freedom in each node).

**Figure 2:**
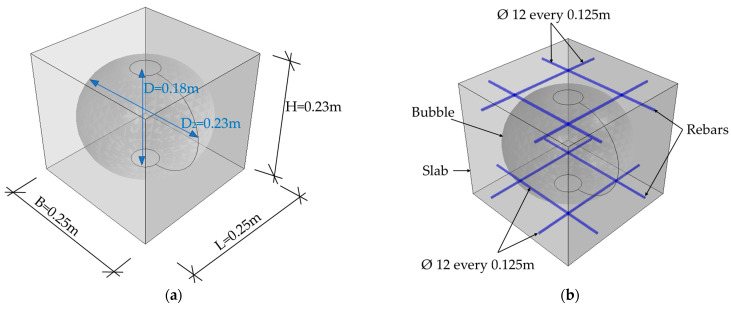
RVE-1—BD230: (**a**) geometry; (**b**) rebars.

In the numerical model 2, a BD230 bubble deck slab was considered. An RVE of 0.25 × 0.25 × 0.23 m (width × length × height) was modeled, see Figure 2a. The plate had an elliptical bubble with dimensions of 0.23 × 0.18 m. The distance between the hole was equal to 0.02 m. As in model 1, the reinforcement was modeled as wire structures and was made of steel, while the plate was a concrete 3D solid structure. The degree of reinforcement of the slab equal to 0.79% in two directions was assumed. In the upper and lower reinforcement mesh, bars with a diameter ϕ=12 mm were spaced every 0.125 cm in two directions (Figure 2b). The concrete slab was divided into a numerical model by using C3D10 elements with dimensions of 0.05 m. The steel bar was meshed with T3D2-type elements. The total number of elements in this model equaled 1866, which gave 3486 nodes and 10,458 degrees of freedom in total.

In the analyzed examples, two materials were used to describe the mechanical behavior of the individual elements of the models: steel and concrete class C30/37. The steel was described by the isotropic linear elasticity relations between stress and strain. For concrete, the linear stress–strain relations were used. In Table 2, the engineering parameters of the materials used in the models are shown, where E is the Young’s modulus, ν is the Poisson′s ratio, and ρ is the density.

### 2.2. Numerical Homogenization Algorithm

**Figure 3:**
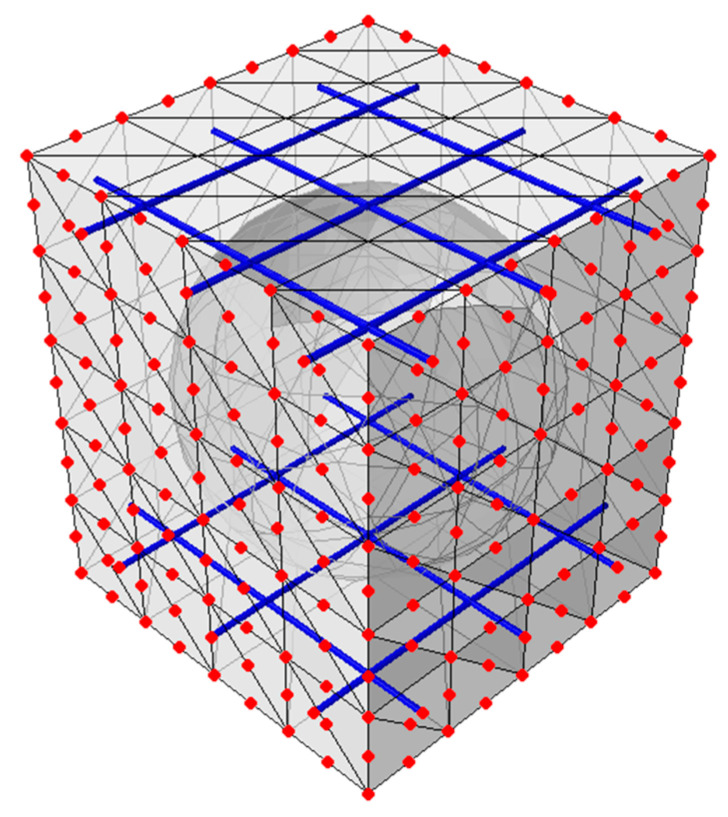
RVE—external (in red color) and internal nodes.

All calculations in this article were made based on the principle of numerical homogenization, which uses the energy equivalence between the full three-dimensional model and the simplified shell model. In the full model, the complete RVE geometry (with all details) is defined, which is then discretized by applying a finite element mesh, and the constitutive relationships for each material are also defined. Since the equivalent stiffness is determined for a certain element whose surface and dimensions are defined by the projection of the RVE onto the x-y surface, in the homogenization process, only the nodes on the vertical RVE surfaces are used (see Figure 3). Therefore, the overall stiffness of the entire model is condensed to those external nodes by using the static condensation method.

The resulting (equivalent) model is reduced to a shell that is described by the tensile, bending, and transversal stiffnesses in both the x and y directions. In this process, both the geometry and the materials (which here are the concrete plate, steel bars, and voids) are homogenized into equivalent structural stiffnesses, on the basis of which, of course, the equivalent material parameters and the effective thickness of such a shell can be calculated. However, in the example analyzed in this paper, this is not necessary; therefore, only the basic steps leading to obtaining equivalent structural stiffnesses will be briefly discussed here.

The generalized displacements at each node on the RVE surface are related to the generalized strains. Therefore, the relationship between the generalized constant strains and the position of the external nodes on the RVE boundary is expressed by the following transformation:

(1) $$u_{i}=W_{i}\;\epsilon_{i},$$

where u is the node displacement vector and ϵ is the strain vector. Here, for a single node ($x_{i}=x$, $y_{i}=y$, $z_{i}=z$), the $W_{i}$ matrix adopted for the RVE model can be easily derived:

(2) $${\left[\begin{array}{c} u_{x}\\ u_{y}\\ u_{z} \end{array}\right]}_{i}={\left[\begin{array}{cccccccc} x & 0 & y/2 & z/2 & 0 & xz & 0 & yz/2\\ 0 & y & x/2 & 0 & z/2 & 0 & yz & xz/2\\ 0 & 0 & 0 & x/2 & y/2 & -x^{2}/2 & -y^{2}/2 & -xy/2 \end{array}\right]}_{i}{\left[\begin{array}{c} \varepsilon_{x}\\ \varepsilon_{y}\\ \gamma_{xy}\\ \gamma_{xz}\\ \gamma_{yz}\\ \kappa_{x}\\ \kappa_{y}\\ \kappa_{xy} \end{array}\right]}_{i},$$

Matrix $W_{i}$ determines the relationship between displacements and effective deformations that are applied to nodes in boundary conditions to which the stiffness of the entire model is condensed. The total energy of elastic deformation is:

(3) $$E=\frac{1}{2}u_{e}^{T}\;K\;u_{e}=\frac{1}{2}\epsilon_{e}^{T}\;W_{e}^{T}\;K\;W_{e}\;\epsilon_{e},$$

where K is the global stiffness matrix. Taking into account the fact that the finite element model is subjected to bending, tension, and transverse shear, for the shell (or plate), the internal energy is:

(4) $$E=\frac{1}{2}\epsilon_{e}^{T}\;W_{k}\;\epsilon_{e}\left\{area\right\},$$

The stiffness matrix for a homogenized composite is easy to extract from a discrete matrix because:

(5) $$W_{k}=\frac{W_{e}^{T}\;K\;W_{e}}{area}.$$

The matrix $W_{k}$ is the ABDR matrix, which can be saved as:

(6) $$A_{k}=\left[\begin{array}{ccc} A_{3\times 3} & B_{3\times 3} & 0\\ B_{3\times 3} & D_{3\times 3} & 0\\ 0 & 0 & R_{2\times 2} \end{array}\right].$$

where A contains the tensile and shear stiffnesses, B contains the combination of tension and bending stiffness, D contains the bending and torsional stiffness, and R contains the transverse shear stiffness.

### 2.3. Sensitivity Analysis

The sensitivity analysis of the bubble deck slabs was examined in terms of changes in the weight of the RVE and its individual stiffnesses, i.e., bending, shearing, and tension/compression stiffness. The parameters of the model were (a) the class of concrete; (b) the height of the slab; (c,d) the dimensions of the bubble or the distance between the holes; and (e,f) the diameter and spacing of the reinforcement. In each subsequent model, one parameter was changed, and the remaining parameters were identical to the reference model (BD340 and BD230). Then, parametric models were used to build the representative volume element that is necessary for numerical homogenization analyses. An individual RVE was built in the same way as the base models described in Section 2.1.

**Figure 4:**
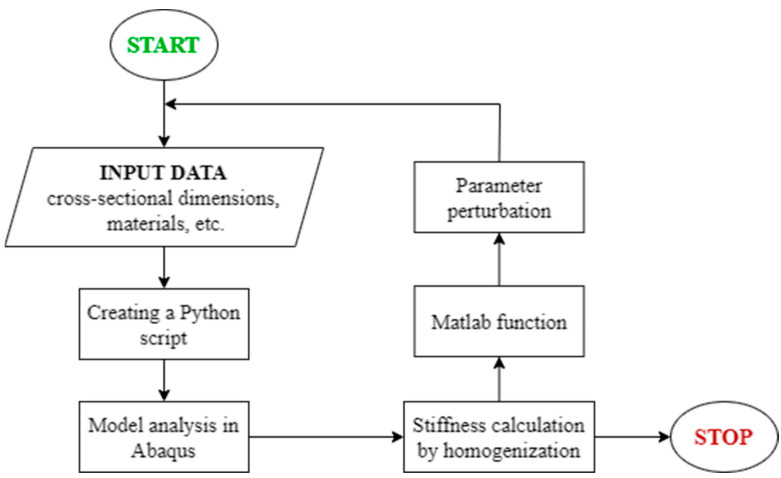
Flowchart of the algorithm for the analysis of sensitivity.

The diagram of the conducted analyses is shown in Figure 4. Although the presented algorithm does not look very complicated, it requires the use of many advanced techniques. After defining the set of input parameters, a python script was launched, and its task was to automatically create any RVE geometry based on the defined parameters. As a reminder, these parameters were concrete class, diameter and spacing of bars, slab height, and diameter and spacing of holes. Then, the defined model was automatically opened in the ABAQUS program, where only the finite element mesh was generated and the global stiffness matrix was assembled. On the basis of the saved data, i.e., node coordinates and the global stiffness matrix, in the next step, static condensation and the $W_{i}$ matrix were computed. These calculations were performed in the MATLAB program, which controlled the whole procedure. On the basis of the calculated $W_{i}$ matrix and the condensed K matrix, the $W_{k}$ matrix was determined (see Equation (6)) for each analyzed geometry. All the scripts written in different environments created a coherent and fully automatic procedure that, based on the input parameters, generated the ABDR stiffnesses of any RVE model, which allowed for the efficient performance of the sensitivity analysis.

## 3. Results

This section presents the results obtained from the analysis of the bubble deck slab using both the numerical homogenization and the sensitivity analysis. The influence of the selected cross-section parameters on the change in the stiffness and weight of the floor was analyzed. The analyses were carried out for two variants of the BD 340 and BD 230 slab. First, the stiffnesses for the reference models described in Section 2.1 were calculated. The results obtained using the homogenization method (Section 2.2) are presented in Table 3. Then, cases with a change in subsequent cross-section parameters were analyzed. The calculated changes in stiffness are presented in the tables with comparison to the reference values.

Table 3 compiles the stiffness submatrices, namely A, D, and the transverse stiffness matrix R (represented in Equation (6)) for both of the RVE reference models.

The slab weight is 5.66 kN/m^2^ for the BD 340 variant and 3.90 kN/m^2^ for the BD 320 variant.

### 3.1. BD 340

This subsection presents the results obtained from the sensitivity analysis based on the numerical homogenization of the bubble ceiling, variant BD340, due to the change in the individual geometrical and material parameters of the cross-section. In each case, only one characteristic of the RVE was changed. Table 4 shows the stiffness results of the BD340 slab obtained as a result of analyses after increasing the selected cross-section or material parameters. The class of concrete was changed to C35/45 (E = 34 GPa), i.e., the Young’s modulus of the material increased by 6.25% compared to the reference value. The perturbation of the other parameters was not the same; for example, the section height (H), hole diameter (D), and rebar spacing were increased by 5.0%, obtaining, respectively, a height equal to H = 0.357 m, a bubble diameter D = 0.284 m, and 0.105 m for the spacing of the rebar. In order to keep realistic values, the diameter of the bars was assumed as *ϕ* = 0.014 m (therefore, the dimension increased by 16.67% compared to the initial RVE model).

When increasing the class of the concrete, the weight of the slab did not change and was equal to 5.66 kN/m^2^. For the other variants of the bubble deck BD340, the weight of the plate changed and was 6.07 kN/m^2^ (for H = 0.357 m), 5.22 kN/m^2^ (D = 0.284 m), 5.75 kN/m^2^ (for ϕ = 0.014 m), and 5.65 kN/m^2^ for bar spacings every 0.105 m.

Table 5 presents the stiffness resulting from the reduction in the individual cross-section and material parameters for the BD340 bubble slab. The Young’s modulus of concrete decreased by 6.25% compared to the reference model (concrete class C25/30—E = 30 GPa was assumed). The height of the cross-section, the spacing of the reinforcement bars, and the diameter of the bubble were, respectively, H = 0.323 m, 0.095 m, and D = 0.256 m. In each of these cases, the given parameter decreased again by 5.0%. In the case of the reinforcement diameter, the reduction was 16.67%, which resulted in using the bars *ϕ* = 0.010 m in the analyses.

In order to better illustrate the influence of a given parameter, the weight of the slab was also calculated. As before, changing the concrete class to C25/30 had no effect on the weight of the slab, and it was 5.66 kN/m^2^. Reducing the section height by 5% reduced the slab weight to 5.25 kN/m^2^. Assuming a smaller diameter of the bars (*ϕ* = 0.010 m), the mass of the bubble plate was equal to 5.59 kN/m^2^, while the reduction in the bubble diameter by 5% compared to the reference model increased the weight of the plate and amounted to 6.05 kN/m^2^. Changing the spacing of the bars (0.095 m) did not significantly affect the weight of the bubble plate, which was 5.67 kN/m^2^.

### 3.2. BD 230

In the next step, the sensitivity analysis based on numerical homogenization was used to compute the BD230 bubble slab type. As for the BD340 variant, only one parameter was increased/decreased. The height was increased by 5% to H = 0.242 m. The dimensions of the elliptical bubble changed to D_1_ = 0.189 m and D_2_ = 0.242 m (increase in dimensions by 5.0% compared to the reference model). The concrete class was changed to C35/45 (increase in Young’s modulus by 6.25%). However, the diameter and spacing of the reinforcing bars increased by 5.0% and 16.67%, respectively, to 0.014 m and 0.131 m. The comparison of the stiffnesses obtained from the plates with a changed selected cross-sectional parameter is presented in Table 6.

The weight of the slab did not change in the case of increasing the concrete class and was equal to 3.90 kN/m^2^. Increasing the dimensions of the holes caused a reduction in the mass of the bubble plate to 3.81 kN/m^2^ (for D_1_ = 0.189 m) and 3.71 kN/m^2^ (for D2 = 0.242 m). For height H = 0.242 m, the weight of the plate was equal to 4.18 kN/m^2^; for reinforcement with diameter ϕ = 0.014 m, the weight was equal to 4.01 kN/m^2^; and the weight was equal to 3.88 kN/m^2^ when the bars were spaced 0.131 m.

The change in stiffness due to a decrease in the value of the selected parameters of cross-sections of the BD230 bubble plate type is presented in Table 7. As previously mentioned, a change in the six parameters was applied, i.e., a change in the concrete class to C25/30 (a reduction in Young’s modulus by 6.25%) and a decrease in the slab height, bubble dimensions, and bar spacing by 5.0%, which resulted in H = 0.219 m, D_1_ = 0.171 m, D_2_ = 0.219 m, respectively, and the distance between the reinforcements being equal to 0.119 m. The diameter of the rods was 0.010 m, which was a decrease in the value of the parameter by 16.67% compared to the initial model.

As before, the change in the concrete class did not affect the value of the slab weight and was exactly the same as in the reference model (3.90 kN/m^2^). Reducing the opening increased the amount of concrete in the element and thus increased the slab weight to 4.00 kN/m^2^ for D_1_ = 0.171 m and 4.09 kN/m^2^ for D_2_ = 0.219 m. The mass of the slab decreased with the change in the ceiling height (H = 0.219 m) and the diameter of the reinforcement ϕ = 0.010 m to the values of 3.63 kN/m^2^ and 3.81 kN/m^2^, respectively. The spacing of the bars equal to 0.119 m caused a slight change in the weight of the slab compared to the initial model and was 3.87 kN/m^2^.

## 4. Discussion

The presented results are limited only to changes in the bending, tension/compression, and transversal shear stiffness of plates with holes due to the perturbations of the selected parameters. The results were obtained for the two types of bubble plates using a sensitivity analysis and numerical homogenization method based on strain energy equivalence. The analyzed slabs had reinforcement and spherical or elliptical bubbles evenly distributed over their entire surface. The conducted analyses allowed us to obtain information on the influence of individual parameters of cross-sections and materials on the plate stiffness for the considered plate. In addition, the influence of the selected parameters on the change in weight was examined. This was a basic step before the optimization of such panels, as it showed which parameters were worth changing and how their change was reflected in the calculated stiffness and weight of the panel.

Table 3 shows the stiffnesses of the bubble plates for the reference models obtained from the sensitivity analysis and numerical homogenization. In turn, Table 4, Table 5, Table 6 and Table 7 present the stiffness of the slabs depending on the change in the parameter, i.e., concrete class, cross-section height, bubble size, diameter of reinforcing bars, and their spacing. The stiffnesses caused by the increase in the values of the individual parameters are shown in Table 4 and Table 6, while those determined after reducing the given feature are shown in Table 5 and Table 7.

For the considered cases, it can be seen that the change in the spacing of the reinforcing bars by 5.0% did not significantly affect the difference in slab stiffness, and we even obtained the same value as in the reference model. Additionally, the diameter of the reinforcement did not affect the shear stiffness, and its effect on the values of compression/tension and bending stiffness was in the range of 2.0–4.0% for the analyzed examples (assuming that the diameter of the reinforcement varied +/−16.67%). The height of the cross-section had the greatest impact on the differences in the obtained results between the reference model and the modified model. When increasing the height of the plate by 5.0%, the bending stiffness increased in the range of about 17.5–19.3% compared to the original model for BD340 and 18.0–20.8% for BD230. For a height reduction of 5.0%, the bending stiffness was reduced by 15.7–17.0% for the first case and 16.0–18.0% for the second case. In addition, it can be seen that the greatest change in tensile/compressive stiffness occurred for different section heights.

The use of a higher class of concrete (C35/45) caused an increase in all stiffnesses by about 5.8–6.6%, while the use of concrete of a lower class (C25/30) reduced the stiffness by about 6.0%. The obtained results showed that the shape of the bubble used in the slab affected the stiffness values in a variable way. Based on the data in Table 3, Table 4, Table 5, Table 6 and Table 7, it can be seen that the spherical bore had the largest change in shear stiffness and the smallest change in bending stiffness. In the case of an elliptical hole, changing the vertical dimension of the bubble affected the stiffness to a lesser extent than its horizontal dimension. The diameter of the reinforcing bars did not change the shear stiffness, and the maximum increase/decrease in bending stiffness was 5% (assuming that the bar diameter changed +/−16.67% compared to the reference model).

In addition, on the basis of the analysis, it can be noted that the change in the class of concrete did not affect the change in the weight of the slab. The greatest impact on the change in the weight of the bubble plate was the increase/decrease in the height of the slab for the analyzed cases and the spherical opening in the case of BD340. A smaller change in the weight of the plate occurred when elliptical bubbles were used than when spherical bubbles were used. The spacing of the bars caused a slight change in the weight of the slab compared to the initial model. In order to make a fair comparison of all the observations presented here, the following normalization procedure was performed. First, the central difference of each change in the measured stiffness value due to the perturbation (decrease and increase) of each analyzed parameter was calculated, and then the results were normalized using the reference value of each stiffness and the actual perturbation step. The normalized sensitivities were computed using the following formula:

(7) $$s_{i}=\frac{R_{i}^{+}-R_{i}^{-}}{h_{i}-\left(-h_{i}\right)}\frac{h_{i}}{R_{i}^{0}}=\frac{R_{i}^{+}-R_{i}^{-}}{2R_{i}^{0}},$$

where $R_{i}^{+}$ is the *i*-th stiffness computed for the *i*-th positive perturbation $h_{i}$, $R_{i}^{-}$ is the *i*-th stiffness computed for the *i*-th negative perturbation $\left(-h_{i}\right)$, and $R_{i}^{0}$ is *i*-th reference stiffness value.

Table 8 and Table 9 present the normalized stiffnesses ADR computed with Equation (7) for the BD340 and BD230 concrete bubble deck slab.

Many researchers recently analyzed the structural, thermal, and acoustic properties of reinforced concrete slabs with balls [38]. In this paper, a procedure is presented by means of which only the structural–strength aspects of hollow plates can be analyzed. Of course, the procedure proposed here can also be extended to an acoustic and thermal analysis of concrete slabs by adding appropriate modules (in Figure 4) in which the necessary analyses would be carried out. General guidelines for the optimal design of reinforced concrete bubble deck slabs can be presented in three main steps: (1) determining the necessary stiffness value for the analyzed floor; (2) specifying additional constraints in the form of, for example, the thermal and acoustic conductivity or the dead weight of the ceiling; and (3) running any optimization algorithm based on the numerical homogenization procedure presented in this paper.

Although the procedure presented in this work has not been experimentally verified, the homogenization method itself, used in the presented procedure, has already been verified on various engineering examples in our previous works [35,36,39,40,41]. The proposed algorithm can of course be implemented in a completely different computing environment; however, the procedure should be consistent with the diagram presented in Figure 4 and the general design guidelines presented in the previous paragraph.

## 5. Conclusions

This sensitivity analysis combined with numerical homogenization can be used in the computer-aided design of optimal bubble deck slabs in construction engineering, etc. The use of the homogenization method adopted for concrete reinforce boards with periodic holes can reduce the calculation time, which is always important in many problems, e.g., strength analysis and slab optimization. Based on the analyses carried out in this work, very important answers were obtained regarding the sensitivity of the basic stiffnesses of the bubble deck slab model and the basic design parameters. The observed relationships clearly indicated that the most important design parameters that play the most important role in the optimization process are the ceiling height and the arrangement and shape of the voids. The class of concrete is twice as less important as the ceiling height, and the class of steel and the diameter of the bars have little effect on the stiffness of the slab cross-section.

The presented algorithm is only the initial step in the optimization procedure, where one can simply change any input parameter and automatically calculate the required stiffness, whether bending or tensile/compressive, along with the change in the basic weight. The algorithmic procedure presented here allows for a quick and comprehensive optimization, which we are going to present in our next article. The sensitivities calculated here will constitute the basic information to appropriately select the optimization method and algorithms, as well as to eliminate the parameters whose impact on individual stiffnesses is negligible.

## Figures and Tables

**Table 2:** Material parameters of steel and concrete used in the tests.

Material	E (GPa)	ν (−)	ρ $\;(\mathrm{kg}/m^{3})$
steel	210	0.3	7900
concrete	32	0.2	2400

**Table 3:** Stiffness matrix for RVE BD340 and BD230 model.

Stiffness	RVE BD340	RVE BD230
A11 ($10^{6}$ MPa mm)	6.85	4.35
A22 ($10^{6}$ MPa mm)	6.85	4.35
A33 ($10^{6}$ MPa mm)	2.48	1.57
D11 ($10^{10}$ MPa mm^3^)	9.92	3.05
D22 ($10^{10}$ MPa mm^3^)	9.92	3.05
D33 ($10^{10}$ MPa mm^3^)	3.58	1.06
R44 ($10^{6}$ MPa mm)	1.68	0.957
R55 ($10^{6}$ MPa mm)	1.68	0.957

**Table 4:** Stiffness for RVE BD340 in case of increasing the value of individual cross-section parameters.

Stiffness	C35/45	H = 0.357 m	D = 0.284 m	ϕ = 0.014 m	Bars Every 0.105 m
A11 ($10^{6}$ MPa mm)	7.25	7.45	6.33	7.04	6.82
A22 ($10^{6}$ MPa mm)	7.25	7.45	6.33	7.04	6.82
A33 ($10^{6}$ MPa mm)	2.64	2.71	2.27	2.48	2.48
D11 ($10^{10}$ MPa mm^3^)	10.48	11.69	9.42	10.28	9.86
D22 ($10^{10}$ MPa mm^3^)	10.48	11.69	9.42	10.28	9.86
D33 ($10^{10}$ MPa mm^3^)	3.81	4.27	3.39	3.58	3.58
R44 ($10^{6}$ MPa mm)	1.79	1.85	1.51	1.69	1.68
R55 ($10^{6}$ MPa mm)	1.79	1.85	1.51	1.69	1.68

**Table 5:** Stiffness for RVE BD340 in case of reducing the value of individual cross-section parameters.

Stiffness	C25/30	H = 0.323 m	D = 0.256 m	ϕ = 0.010 m	Bars Every 0.095 m
A11 ($10^{6}$ MPa mm)	6.45	6.30	7.40	6.70	6.92
A22 ($10^{6}$ MPa mm)	6.45	6.30	7.40	6.70	6.92
A33 ($10^{6}$ MPa mm)	2.33	2.27	2.70	2.48	2.48
D11 ($10^{10}$ MPa mm^3^)	9.35	8.36	10.31	9.65	9.97
D22 ($10^{10}$ MPa mm^3^)	9.35	8.36	10.31	9.65	9.97
D33 ($10^{10}$ MPa mm^3^)	3.36	2.97	3.74	3.58	3.58
R44 ($10^{6}$ MPa mm)	1.58	1.55	1.84	1.68	1.69
R55 ($10^{6}$ MPa mm)	1.58	1.55	1.84	1.68	1.69

**Table 6:** Stiffness for RVE BD230 in case of increasing the value of individual cross-section parameters.

Stiffness	C35/45	H = 0.242 m	D_1_ = 0.189 m	D_2_ = 0.242 m	ϕ = 0.014 m	Bars Every 0.131 m
A11 ($10^{6}$ MPa mm)	4.60	4.76	4.20	4.07	4.51	4.31
A22 ($10^{6}$ MPa mm)	4.60	4.76	4.20	4.07	4.51	4.31
A33 ($10^{6}$ MPa mm)	1.66	1.72	1.50	1.46	1.57	1.57
D11 ($10^{10}$ MPa mm^3^)	3.22	3.60	2.95	2.97	3.21	3.00
D22 ($10^{10}$ MPa mm^3^)	3.22	3.60	2.95	2.97	3.21	3.00
D33 ($10^{10}$ MPa mm^3^)	1.13	1.28	1.03	1.03	1.06	1.06
R44 ($10^{5}$ MPa mm)	10.16	10.47	9.41	8.49	9.60	9.57
R55 ($10^{5}$ MPa mm)	10.16	10.47	9.41	8.49	9.60	9.57

**Table 7:** Stiffness for RVE BD230 in case of decreasing the value of individual cross-section parameters.

Stiffness	C25/30	H = 0.219 m	D_1_ = 0.171 m	D_2_ = 0.219 m	ϕ = 0.010 m	Bars Every 0.119 m
A11 ($10^{6}$ MPa mm)	4.10	3.95	4.51	4.64	4.24	4.40
A22 ($10^{6}$ MPa mm)	4.10	3.95	4.51	4.64	4.24	4.40
A33 ($10^{6}$ MPa mm)	1.47	1.41	1.63	1.67	1.57	1.57
D11 ($10^{10}$ MPa mm^3^)	2.88	2.56	3.14	3.13	2.93	3.09
D22 ($10^{10}$ MPa mm^3^)	2.88	2.56	3.14	3.13	2.93	3.09
D33 ($10^{10}$ MPa mm^3^)	0.998	0.869	1.10	1.09	1.06	1.06
R44 ($10^{5}$ MPa mm)	8.98	8.77	9.74	10.66	9.55	9.57
R55 ($10^{5}$ MPa mm)	8.98	8.77	9.74	10.66	9.55	9.57

**Table 8:** Normalized stiffness for RVE BD340.

Stiffness	C25/30	H = 0.323 m	D = 0.256 m	ϕ = 0.010 m	Bars Every 0.095 m
A11 (%)	5.84	8.39	−7.81	2.48	−0.73
A22 (%)	5.84	8.39	−7.81	2.48	−0.73
A33 (%)	6.25	8.87	−8.67	0.00	0.00
D11 (%)	5.70	16.78	−4.49	3.18	−0.55
D22 (%)	5.70	16.78	−4.49	3.18	−0.55
D33 (%)	6.28	18.16	−4.89	0.00	0.00
R44 (%)	6.25	8.93	−9.82	0.30	−0.30
R55 (%)	6.25	8.93	−9.82	0.30	−0.30

**Table 9:** Normalized stiffness for RVE BD230.

Stiffness	C25/30	H = 0.219 m	$D_{1}\;=\;0.171\;m$	$D_{2}\;=\;0.219\;m$	ϕ = 0.010 m	Bars Every 0.119 m
A11 (%)	5.75	9.31	−3.56	−6.55	3.10	−1.03
A22 (%)	5.75	9.31	−3.56	−6.55	3.10	−1.03
A33 (%)	6.05	9.87	−4.14	−6.69	0.00	0.00
D11 (%)	5.57	17.05	−3.11	−2.62	4.59	−1.48
D22 (%)	5.57	17.05	−3.11	−2.62	4.59	−1.48
D33 (%)	6.23	19.39	−3.30	−2.83	0.00	0.00
R44 (%)	6.17	8.88	−1.72	−11.34	0.26	0.00
R55 (%)	6.17	8.88	−1.72	−11.34	0.26	0.00

## Data Availability

Not applicable.
